# Questions and Answers Related to the Prebiotic Production of Oligonucleotide Sequences from 3′,5′ Cyclic Nucleotide Precursors

**DOI:** 10.3390/life11080800

**Published:** 2021-08-08

**Authors:** Judit E. Šponer, Jiří Šponer, Aleš Kovařík, Ondrej Šedo, Zbyněk Zdráhal, Giovanna Costanzo, Ernesto Di Mauro

**Affiliations:** 1 Institute of Biophysics of the Czech Academy of Sciences, Královopolská 135, 61265 Brno, Czech Republic; sponer@ncbr.muni.cz (J.Š.); kovarik@ibp.cz (A.K.); 2 Central European Institute of Technology, Masaryk University, Kamenice 5, 62500 Brno, Czech Republic; ondrej.sedo@ceitec.muni.cz (O.Š.); zdrahal@sci.muni.cz (Z.Z.); 3 Institute of Molecular Biology and Pathology, CNR, Piazzale A. Moro 5, 00185 Rome, Italy; giovannamaria.costanzo@cnr.it (G.C.); ernesto.dimauro@uniroma1.it (E.D.M.)

**Keywords:** polymerization, cyclic nucleotides, prebiotic chemistry

## Abstract

Template-free nonenzymatic polymerization of 3′,5′ cyclic nucleotides is an emerging topic of the origin of life research. In the last ten years, a number of papers have been published addressing various aspects of this process. These works evoked a vivid discussion among scientists working in the field of prebiotic chemistry. The aim of the current review is to answer the most frequently raised questions related to the detection and characterization of oligomeric products as well as to the geological context of this chemistry.

## 1. Introduction

**Figure 1:**
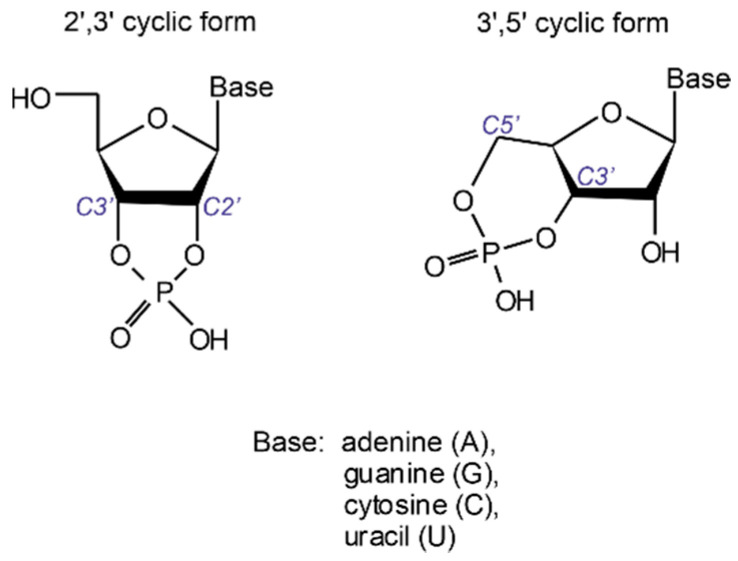
Structural formulas of 3′,5′ and 2′,3′ cyclic nucleotides.

Production of oligonucleotide sequences from nucleotide precursors in the absence of enzymes and template molecules is one of the most challenging problems of modern origin of life research. 2′,3′ cyclic nucleotides (see Figure 1) have long been considered as potent precursors of the most ancient oligonucleotide sequences formed on our planet [1,2,3,4]. This motivated numerous attempts dealing with the reconstruction of their synthesis under plausible prebiotic conditions [5,6,7,8]. Nonetheless, nucleotides containing a phosphodiester linkage confined in a strained 5-membered ring are unstable in an acidic environment and undergo a quick ring-opening reaction on the timescale of minutes [9].

In contrast, the 3′,5′ cyclic form possesses sufficient stability to withstand extreme conditions as conferred by low-pH environments, most likely ubiquitous on the early Earth [10,11]. Partly because of its significance played in modern biology (cGMP and cAMP are cofactors of key biochemical reactions [12]) and partly because of its unique aggregation properties, 3′,5′ cyclic guanosine monophosphate (hereafter 3′,5′ cGMP, the rest of the abbreviations used are explained at the end of the paper) was the target of a series of studies aimed at unraveling the process that could lead to the formation of the first oligonucleotide sequences on the early Earth [13,14,15,16,17,18].

The concept of polymerization studies using 3′,5′ cyclic nucleotides is strikingly different from that of preceding works published on prebiotic polymerization processes, which set a goal to find high-yielding chemical routes to oligonucleotides. These latter studies (for an overview see e.g., [19]) use highly activated monomers, because they enable achieving higher polymerization yields. Nevertheless, the lower stability of highly activated precursors (e.g., phosphorimidazolides, nucleotide triphosphates or 2′,3′ cyclic nucleotides) raises questions regarding their accumulation and long-term survival in the prebiotic pool.

Polymerization studies targeting 3′,5′ cyclic nucleotides place a special emphasis on identifying the conditions that are compatible with a sustainable oligonucleotide production on longer time scales in a geochemical context relevant to the early Earth. This requires not only sufficiently stable monomers, but also a robust chemistry. As Albert Eschenmoser suggested [20], the robustness of a prebiotic synthetic network lies in the multimodality of catalytically accessible chemical pathways between reactants and final products, because it enables adaptation of the chemical system to the quickly changing chemical environment.

In the current paper we provide a brief account of the available knowledge on the polymerization of 3′,5′ cyclic nucleotides. Since this research has been the focus of general attention for a long time, a number of questions and objections have been raised by the prebiotic chemistry community related to this topic. Our main goal with this paper is to provide an in-depth answer to the most frequent ones and to clarify those disputed points, which emerged after publishing the original studies.

## 2. How Could 3′,5′ Cyclic Nucleotides Accumulate in the Prebiotic Pool?

Synthesis of 2′,3′ cyclic nucleotides [5,6,7,8] was demonstrated by a number of studies both in aqueous and non-aqueous media. In contrast, no attention has so far been devoted to the prebiotic synthesis of 3′,5′ cyclic nucleotides. Nevertheless, based on historical studies by Khorana et al., a simple potential prebiotic route can be outlined to these compounds: Reference [21] illustrates that in the presence of carbodiimides nucleoside 5′-phosphates can be converted into the 3′,5′ cyclic form. Since carbodiimides form in large amounts upon thermal treatment of formamide in the presence of meteorites [22] and nucleoside-5′-phosphates belong to the main products of phosphorylation reactions conducted in formamide [7], accumulation of 3′,5′ cyclic nucleotides seems to be plausible in a formamide-rich medium under prebiotic conditions. Work on this topic is in progress.

## 3. Mechanism of Ring-Opening Polymerization Reactions Involving 3′,5′ Cyclic Nucleotides

Ring-opening polymerization reactions of cyclic phosphate and phosphonate esters in non-aqueous medium are well-known in the literature [23,24]. These reactions are commonly catalyzed by DBU suggesting that a base-catalyzed ring-opening polymerization operates in this case. In the first reports on polymerization of 3′,5′ cGMP the highest reaction yields were observed at pH 9 achieved with Tris-HCl buffering suggesting a base-catalyzed reaction mechanism [14]. Addition of DBU also promoted the reaction [14]. Recent studies on the pH-dependence of the reaction conducted in dry state indicated that the reaction proceeds by an acid- rather than a base-catalyzed mechanism [25]. We note, that this observation does not contradict the original observation made in a pH = 9 Tris-HCl buffer, since a recent study showed that the pH of this buffer exhibits an extraordinarily strong temperature dependence [26]. Thus, the actual pH of the Tris-HCl buffer could be 3–4 orders of magnitude lower at the temperature of the polymerization experiment (75–85 °C). At this temperature, a large part of the water present in the buffer (commonly only 15 μl is added to the dry material) evaporates and condenses in the tip of the test tube, thus, the pH-shift towards acidic becomes even more pronounced, making the acid-catalyzed mechanism more plausible [25].

**Figure 2:**
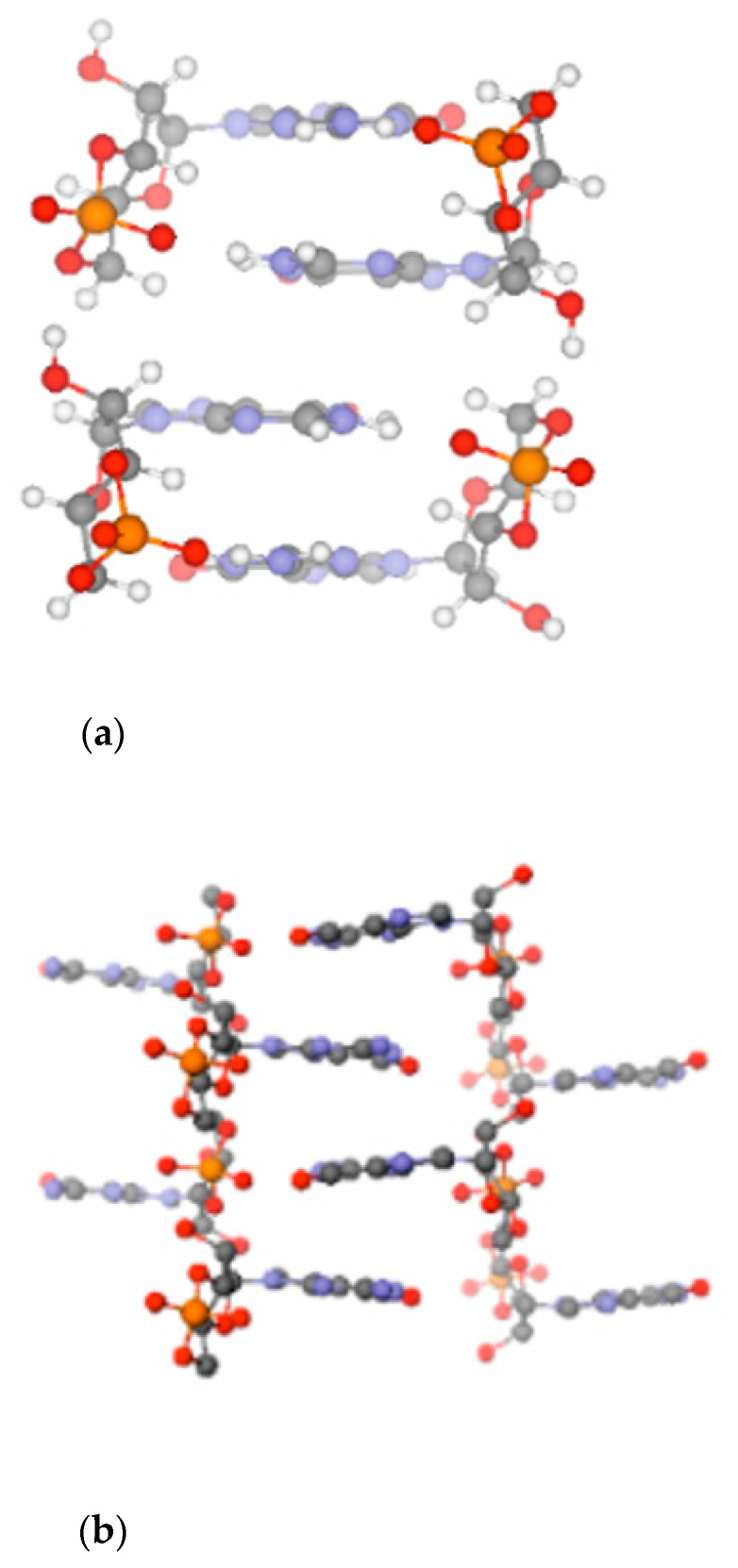

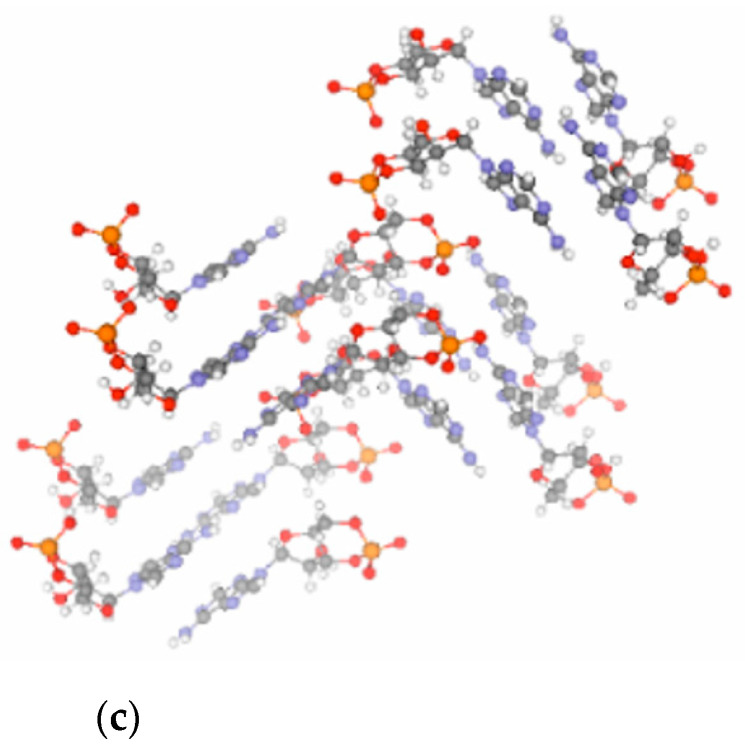
Theoretically predicted optimum model for the transphosphorylation of 3′,5′ cGMP [16] (**a**) as well as crystal structures of 3′,5′ cGMP [28] (**b**) and 3′,5′ cAMP [29] (**c**). Color coding: C—grey; O—red; N—blue; P—orange; H—white.

**Figure 3:**
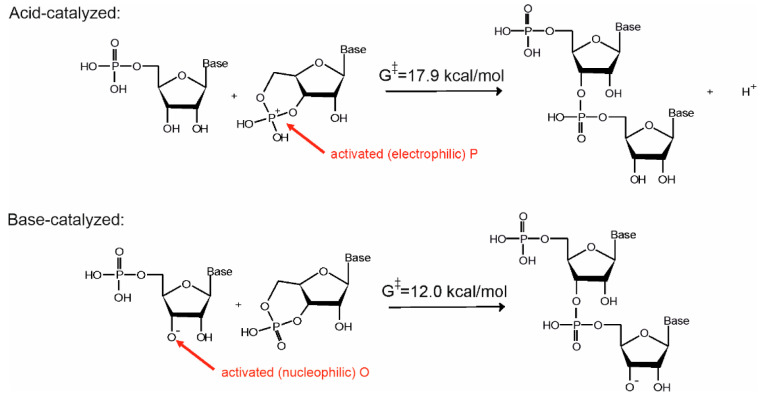
Mechanistic models for the chain-extension step of the ring-opening polymerization of 3′,5′ cGMP. G^‡^ refers to the computed activation free energy of the reaction step [16,25].

A computational investigation has shown that a ladder-like stacked supramolecular architecture may host both the base- as well as the acid-catalyzed reaction routes (see Figure 2a) [16]. The only difference between the two pathways is that while in the base-catalyzed reaction pathway the nucleophile is activated by deprotonation of the ribose O3′ position, in the acid-catalyzed pathway the phosphate of the substrate is activated by protonation (see Figure 3). Let us note that use of both activation strategies is widespread in organic chemistry to facilitate addition-elimination-type substitution reactions [27].

Amazingly, the stacked structure of 3′,5′ cGMP predicted by state-of-the art electronic structure (QM) calculations is essentially identical to the stacking pattern seen in the X-ray structure (see Figure 2b), reflecting the unique capability of 3′,5′ cGMP to self-associate by stacking. The electronic structure calculations in addition without doubt prove that this robust ground-state stacking arrangement is readily capable to support a reactive geometry for the oligomerization process. In view of this we conclude that all claims that 3′,5′ cGMP cannot adopt a reactive conformation, etc., should be dismissed as scientifically unfounded. Due to its stacking properties 3′,5′ cGMP can clearly approach a reactive conformation for polymerization on its own and does not need help from any other midwife molecules or activating agents. The ability of biopolymers and their monomers to self-assemble into reactive geometries could play a pivotal role at the emergence of life on our planet [30].

Our computations show that both mechanisms have similar activation energies and thermodynamics [16,25]. Thus, both mechanisms are chemically plausible per see. Nevertheless, the acid-catalyzed pathway seems to be more likely in a prebiotic context. This is because the reaction requires formation of a well-defined supramolecular architecture prior to the transphosphorylation reactions which is more easily achievable under acidic conditions.

It has been found that the crystal structure [28,31] of free acid- and Na-form cGMP provides optimum steric conditions for the transphosphorylation reactions necessary to form intermolecular phosphodiester linkages between the cyclic monomers (see Figure 2b) [16]. Notably, the crystal structure of cAMP [29] (Figure 2c) is less suited to support the polymerization chemistry. In line with this, only short (3–4 nt long) oligomers form from 3′,5′ cAMP in contrast to 3′,5′ cGMP, which polymerizes to a length up to 15–20 nucleotides. In addition, polymerization of 3′,5′ cAMP is considerably slower as compared to that of 3′,5′ cGMP [32].

While the computed thermodynamic and kinetic parameters are very similar for the acid- and base-catalyzed mechanisms, crystallization of the cGMP monomers under acidic and basic conditions is strikingly different [18,25]. While the H-form (i.e., acidic) monomers crystallize on the timescale of minutes, crystallization of the Na-form (i.e., basic salt) material requires several days [31] due to the apparent electrostatic repulsion between the negatively charged cGMP^-^ monomers. Faster crystallization of the acid-form cGMP molecules enables faster formation of the stacked supramolecular architecture that is the prerequisite of the polymerization reaction. This is compatible only with a low pH environment and an acid-catalyzed mechanism [25].

## 4. Is Detection of Oligonucleotides Formed from 3′,5′ cGMP by Denaturing Gel Electrophoresis Biased by Noncovalent Adducts?

A number of methods have been used for detection of the various products formed in the oligomerization of 3′,5′ cGMP. Among them, electrophoresis on denaturing gels has been the most frequently applied technique mainly because of its high sensitivity. The method was used in combination with radioactive [13,14,16,17,18,32] as well as fluorescent labeling [15,25] (for a recent detailed description of methodological details see [18,25]).

**Figure 4:**
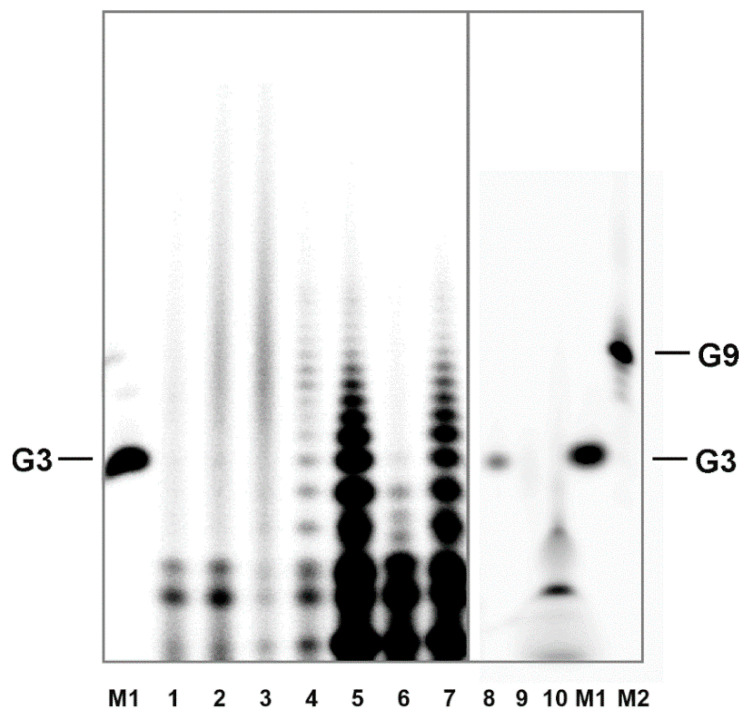
Control experiments with variously treated 3′,5′ cGMP as well as with guanosine and guanosine 5′-triphosphate (GTP) adopted from [18]. Lane 1: 150 μl 1 mM cGMP-H freeze-dried in an Eppendorf tube (for ~1 h) without subsequent thermal treatment at elevated temperatures. Lane 2: 150 μl 1 mM cGMP-H freeze-dried in an Eppendorf tube (for ~1 h) and treated at 80 °C for 5 min. Lanes 3 and 4: the same as lane 2, except that the thermal treatment at 80 °C was performed for 15 and 60 min, respectively. Lane 5: 66 × 3 μl of 1 mM cGMP-H solution deposited in a dropwise manner on the bottom of a preheated (80 °C) borosilicate beaker. The next drop was always deposited on the same place after drying of the previous one. The dry material was treated at 80 °C for additional 5 h. Lane 6: 6 × 7.5 μl of 1 mM cGMP-H solution was deposited in a dropwise manner into 5 spots on the bottom of a preheated (80 °C) borosilicate beaker. The next drop was always deposited on the same place after drying of the previous one. The dry material was treated at 100 °C for additional 5 h. Note that the optimum temperature for the polymerization reaction is ~75–85 °C, according to [14] and [16], i.e., this experiment was performed outside the optimum reaction temperature range. Lane 7: 30 × 6.6 μl of 1 mM cGMP-H solution deposited in a dropwise manner on the bottom of a preheated (80 °C) borosilicate beaker. The next drop was always deposited on the same place after drying of the previous one. The dry material was treated at 80 °C for additional 5 h. Lane 8: 150 μl of 2 mM (~300 ng) G3 oligomer (from Biomers) was freeze-dried in an Eppendorf tube, followed by a heat-treatment at 80 °C for 3 h. Lane 9 (almost empty): 150 μl of 1 mM guanosine freeze-dried in an Eppendorf tube and subsequently treated at 80 °C for 5 h. Since guanosine has the same aggregation properties as 3′,5′ cGMP-H, absence of oligomerization products shows that the kinase used for phosphorylation does not recognize non-covalent aggregates of monomers. Lane 10: 150 μl of 1 mM GTP prepared and treated as the guanosine sample (diffuse smeared bands towards the bottom are likely caused by the highly charged character of the molecule). Samples were precipitated using the procedure given in [18]. M1 = G3 (Biomers) marker. M2 = G9 (Biomers) marker.

The radioactive labeling has often been criticized because of the use of enzymes and the necessity of a precipitation step that is used to remove the excess ^32^P-ATP prior to loading the labeled material on the gel. Potentially, this could lead to the formation of non-covalent aggregates. This possibility has been tested with a simple experiment, in which guanosine has been treated in the same way as 3′,5′ cGMP [18]. Aggregation properties of H-form 3′,5′ cGMP are very similar to those of guanosine. Thus, it is reasonable to expect, that if aggregates formed from guanosine withstand the precipitation and labeling procedure, they would have to produce multiple band structures on the denaturing gel, similar to those of the oligomerization products. As Figure 4 illustrates, this has not been observed. Further, this experiment excludes the possibility that the polynucleotide kinase used for phosphorylation could induce oligomer formation.

Denaturation PAGE is standardly run in the presence of 50% urea at elevated temperatures which effectively disrupts intermolecular bonds. Together, detection of non-covalent aggregates can be excluded. The fact that the oligomers are efficiently labeled in a kinase reaction suggests that a considerable portion of the polymerization products contains free 5′-OH groups.

## 5. Is Mass Spectrometric Detection of Oligonucleotides Formed from Cyclic Nucleotide Precursors Biased by Non-Covalent Adducts?

A frequently used methodology for oligonucleotide detection is MALDI-ToF mass spectrometry. The approach was used for detection of oligonucleotides formed from cyclic nucleotide precursors as well. Nevertheless, soon after its first use it has been shown that this method is often biased by non-covalent adduct formation, since the molar weight of adducts consisting of *n* cyclic monomers equals to that of an *n*-mer oligomer having a 2′,3′ cyclic end [33]. Likewise, molecular weight of an *n*-mer oligomer is the same as that of *n*-monomers plus a water molecule.

Studying the fragmentation spectra of the species detected with MALDI MS could be one of the potential remedies for this problem. For example, inspection of the polymerization products formed from 3′,5′ cAMP allowed a successful identification of pApA dimers. The fragmentation spectra clearly revealed signals corresponding to pA as well as pAp fragments, which exhibited roughly the same intensities as those found in the fragmentation spectra of pApA standards [32]. Unfortunately, the same method cannot be used for identifying pGpG products, since intensity of the pG signal, which also corresponds to the hydrolyzed monomer, is an order of magnitude higher than that of the more important pGp fragment.

In our studies dealing with the polymerization of 3′,5′ cCMP [34], we used ESI-MS detection, which (at least for this monomer) was less biased by formation of non-covalent adducts. In particular, in the spectrum of the heat-treated sample, we did not observe the peak shifted by +18 Da, corresponding to the adduct of the monomer with water or hydrolyzed monomer, whereas the signal corresponding to the pCpC covalent product was observed. Fragmentation of this signal produced the expected pCp and pC species, verifying the covalent character of the pCpC dimer.

**Figure 5:**
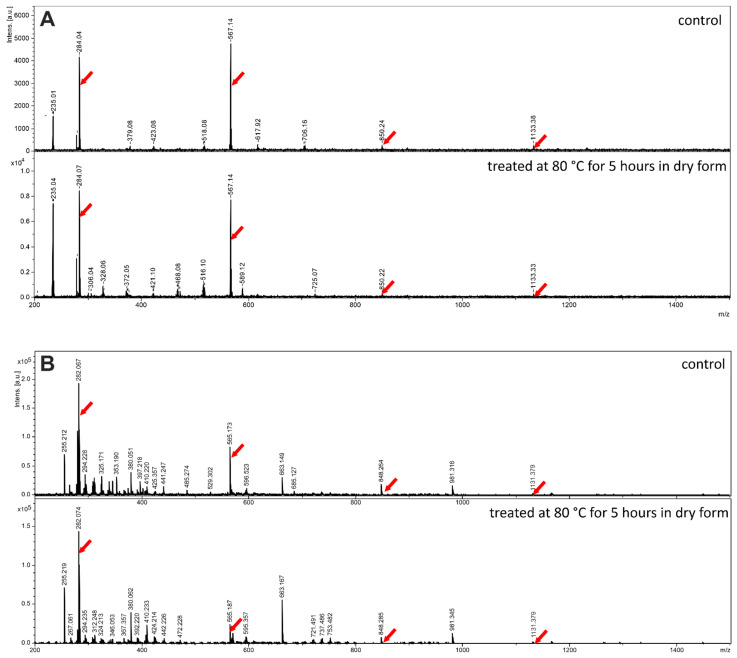
MALDI-ToF MS (measured in reflectron positive detection mode, panel (**A**)) and ESI-MS (measured in negative ion mode, panel (**B**)) spectra of untreated and heat-treated guanosine samples. The heat treatment was performed in dry state at 80 °C for 5 h. Signals of guanosine and its adducts are indicated with an arrow.

To assess the extent of non-covalent adduct formation in the MALDI-ToF MS and ESI-MS spectra of polymerization products formed from guanine-containing monomers, we investigated the spectra of guanosine, a molecule with remarkably similar aggregation properties as that of 3′,5′ cGMP. Figure 5 shows that in contrast to the ESI-MS detection of oligoC sequences discussed in [34], non-covalent guanosine aggregates up to tetramer are observed both by MALDI-ToF as well as by ESI mass spectrometry. This is likely caused by the outstandingly high propensity of guanine-derivatives to form stacked aggregates independently on the medium. On the other hand, as expected, in lack of covalent oligomer formation, there is almost no difference between the spectra of the untreated and heat-treated (conducted at 80 °C for 5 h in dry form) materials.

## 6. What Kind of Other Detection Methods Support Oligonucleotide Formation from 3′,5′ cGMP?

In addition to detection by electrophoresis and mass spectrometry, the covalent nature of the oligomers formed in the polymerization of 3′,5′ cGMP has been studied by a number of other methods (for a summary of methods applied for characterization of products formed from 3′,5′ cyclic nucleotides, see Table 1).

In [14] ^31^P-NMR spectroscopy showed that upon heat treatment of 3′,5′ cGMP new signals appear in the 0 to −2.6 chemical shift range, characteristic to RNA oligonucleotides. Since the signal of phosphorus of acyclic or 2′,3′ cyclic monomers falls into the positive chemical shift range, the new signals resulting from sample treatment have been attributed to RNA-oligomers formed from 3′,5′ cyclic precursors.

Infrared nanospectroscopy has been used to follow the change of the IR spectrum of a 3′,5′ cGMP sample deposited on gold surface upon heat-treatment at 80 °C [17]. The analysis has shown that a new band appears at 970 cm^−1^, i.e., in the fingerprint region characteristic to RNA-backbones, in the spectrum of the heat-treated material.

A recent study combines HPLC-separation and mass spectrometry to identify oligonucleotide products formed when polymerizing 3′,5′ cGMP [25]. This investigation has shown that besides non-covalent aggregates a noticeable amount of covalently bound oligomers is formed.

In summary, although the individual experimental methods used to monitor the oligomerization of 3′,5′ cNMP compounds are complicated by some uncertainties, on aggregate, the 3′,5′ cNMP polymerization reactions have been characterized by multiple independent methods and are thus more thoroughly scrutinized than many other oligomerization processes discussed in contemporary prebiotic chemistry literature.

## 7. Does the Oligomerization Reaction of 3′,5′ cGMP Proceed in the Presence of Water?

The reaction was studied under a multitude of experimental conditions. Its temperature optimum was found to be ca. 80 °C [13,14,16]. It was found that at least one drying step is necessary to get oligonucleotides longer than trimers [15,17]. Oligomer formation is regularly observed after ca. 1 h of reaction time. The reaction yields exhibit an oscillatory behavior on longer (several days) time scales, regardless of whether the reaction is conducted in dry state or in a nearly saturated solution [18]. The oscillatory behavior of the reaction has pointed at the importance of phase-separation processes in this chemistry. Nonetheless, oligomer (>trimer) formation is detectable on timescales of several weeks, assumed that at least partial drying of the monomers occurs in the course of sample treatment.

Thus, in summary, our investigations show that the presence of water in the reaction mixture is not poisonous for the polymer formation if part of the material is at least temporarily present in dry form during the sample treatment. Nonetheless, only short (≤3 nt long) oligomers form if drying of monomers is prevented [25,35].

## 8. Technical Requirements of the Oligomerization Experiment

When studying the polymerization of 3′,5′ cGMP, it was found that the drying process itself induces oligomer formation. Thus, conducting the polymerization experiment in a straightforward manner requires the use of a specially purified material that was never precipitated or dried during the sample preparation procedure. Using material purchased in dry form, due to its oligonucleotide content, produces false positive results.

When working with minerals, the specimens must be thoroughly cleaned by using H_2_O_2_, ethanol to remove their organic material content, which may degrade the oligomers formed in the polymerization reaction.

## 9. How Much is the Polymerization of 3′,5′ cGMP Sensitive to the Presence of Cations? May It Be Relevant in a Geological Context?

Earlier reports interpreted the absence of polymer formation from an Na-form salt of 3′,5′-cGMP by the fact that cations block the anionic ring-opening polymerization mechanism [16]. A recent study revisited this issue, and showed that presence of cations in the reaction mixture is not as restrictive as thought before [25]. As long as the polymerization is performed in an acidic environment, i.e., the monomers are neutral molecules, cations start inhibiting the reaction at very large (ca. 50×) excess. This means that basic pH rather than cations exert an inhibitory effect on the reaction [25].

**Figure 6:**
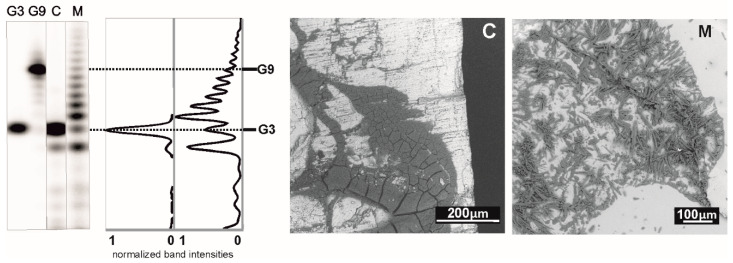
3′,5′ cGMP crystallizes and polymerizes on muscovite mica (M). No crystallization and polymer formation are observed on calcite (C). From left to right: electrophoretic analysis of the polymerization products formed by heat-treatment (80 °C, 5 h) of the dry material deposited in a dropwise manner on the mineral surface; densitometric analysis of the electrophoretic images; electron microscopic images of the dry materials deposited on the mineral surfaces. Adopted from [18].

The argument that the polymerization reaction is sensitive to cations logically suggested that such chemistry might be irrelevant to a real prebiotic environment, where free cations were likely available in large amounts. To project laboratory experiments into more geologically plausible models, we recently tried deposition of the cGMP monomers from a dropping solution on various heated mineral surfaces relevant to an early Earth scenario. The minerals chosen for our experiments were all included in Hazen’s “preliminary species list” [36]. We found that minerals, like amorphous and crystalline silica forms, mica, amphibole and other silicates, like andalusite, may host the reaction. On the other hand, minerals that chemically react with the acid-form of 3′,5′ cGMP leading to its deprotonation (like carbonate- and serpentine-group minerals or olivine) are incompatible with the chemistry [18]. This way the free acid form of 3′,5′ cGMP is converted into a salt-form material, which does not crystallize on the time scales of the experiment. The lack of crystal order in the dry deposited material then interferes with the oligonucleotide formation (see Figure 6).

In summary, the ions per se do not prevent the oligomerization process.

## 10. Does Depurination Hamper Formation of Short Oligonucleotide Sequences from 3′,5′ cGMP?

Higher propensity of guanine-containing nucleotides to depurinate in acidic conditions is well known [37]. Nevertheless, depurination reactions also require the presence of water in the reaction mixture. Under drying conditions, where oligonucleotide formation has been demonstrated, the depurination process is not significant and is unable to outcompete the polymerization reaction. It does not interfere with the oligomerization. Our recent mass spectrometric investigation (using both ESI- and MALDI-ToF MS methods) focused on the low molecular weight fraction products present in the reaction mixture and has shown that sample treatment in dry form at 80 °C for 5 h results only in a negligible depurination of the cyclic monomers [18]. In contrast, under dry-wet cycling conditions used by Rajamani et al. [38], half-life of acyclic 5′ AMP was estimated to be 6.5 h only. This study suggests that repeated dry-wet cycles may interfere with formation of oligonucleotides from purine nucleotide precursors.

We note, that depurinated short oligonucleotide sequences have been detected among the polymerization products by gel-electrophoresis [18]. Their origin is not necessarily associated with the polymerization process, since they could be the result of the sample treatment required by the radioactive end labeling and the denaturing gel electrophoresis. Signals of depurinated oligomer products were not observed in the MALDI-ToF MS analysis presented in [16].

## 11. Conclusions: What are the Potential Reasons for the Erratic Reproducibility of 3′,5′ cGMP Polymerization Experiments?

Lane et al. [39] rightfully designated reproducibility of the 3′,5′ cNMP oligomerization reactions as “erratic”. This means that the reaction requires suitable physical and chemical conditions to proceed, and if these conditions are not fulfilled, the reaction is inhibited, which can lead to the erroneous claim that the experiments could not be reproduced. However, erratic behaviors of chemical processes are not uncommon (on example could be crystallization processes which often require many empirical trial and error experiments to find the right condition) and have nothing to do with reproducibility. In fact, some degree of erratic behavior may be advantageous in chemical evolution, as it indicates the reaction is variable and sensitive to external conditions. Therefore, it can be regulated and optimized by external factors, and coupled with other reactions. In other words, while we certainly found some suitable conditions upon which the 3′,5′ cGMP oligomerization occurs, we almost certainly did not find the optimal conditions. Importantly, this polymerization reaction does not seem to need a sophistically tailored chemical environment with other supportive chemical species; it appears to be responsive to straightforward changes in common physical-chemistry conditions.

Apart from trivial reasons (like not ensuring conditions in which relatively fragile short RNA sequences are capable of surviving; or using a salt form of the cyclic nucleotide monomer), over the years we identified several other reasons that might potentially hamper detection of oligonucleotides formed from 3′,5′ cGMP in specific experimental conditions.

We showed that the reaction itself exhibits an oscillatory behavior on longer time scales [17]. This feature originates in the highly non-equilibrium nature of this chemistry and manifests itself as the strong time dependence of the reaction yields. For this reason, if the reaction time is improperly selected for a given experiment, only low amounts of short (≤3 nt long) oligomers may be detected. Thus, it is advisable to study the reaction on longer time scales at several time points for a successful detection.

Another very important feature of the reaction is that at least one drying step is needed for a continued oligonucleotide production. This means that to a lesser extent, longer (>3 nt) oligonucleotides do form even in the presence of water, if the experimental setup allows for partial drying of the sample. In our previous studies we showed that this can be achieved by rewetting of the dry material, or by partial drying of the sample during the heat treatment [17,25].

The method used for drying may also influence the outcome of detection for two reasons. Prolonged (several hours long) drying in a low-performance vacuum evaporator may cause depurination of the nucleotide monomers. Further, too fast drying at elevated temperatures may occasionally lead to the formation of a glassy, non-crystalline material with randomly placed monomers even when using an H-form material. Obviously, in this case the lack of a properly organized supramolecular architecture interferes with the oligonucleotide formation. Many doubts about the reproducibility of the cGMP oligomerization reaction may stem from variability between experiments leading to different amounts and quality of crystalline materials.

## Figures and Tables

**Table 1:** Summary of the methodologies used for detection of oligonucleotides formed from 3′,5′ cyclic nucleotide precursors.

Method	Monomer	References
Denaturing gel-electrophoresis with ^32^P-labelling	3′,5′ cGMP 3′,5′ cAMP	[13,14,16,17,18] [32]
Denaturing gel-electrophoresis with intercalating fluorescent labelling	3′,5′ cGMP	[15,25]
MALDI-ToF	3′,5′ cGMP 3′,5′ cAMP 3′,5′ cCMP	[14,15,16] [32] * [34]
ESI-MS	3′,5′ cCMP	[34] *
HPLC-ESI-MS	3′,5′ cGMP	[25]
^31^P-NMR	3′,5′ cGMP	[14]
Infrared nanospectroscopy	3′,5′ cGMP	[17]

* Including MS/MS fragmentation analysis.

## Data Availability

Data reported in this paper can be obtained from the authors upon request.

## Abbreviations

3′,5′ cGMP	3′,5′ cyclic guanosine monophosphate
3′,5′ cGMP-H	free adic form of 3′,5′ cyclic guanosine monophosphate
3′,5′ cAMP	3′,5′ cyclic adenosine monophosphate
3′,5′ cCMP	3′,5′ cyclic cytosine monophosphate
5′ AMP	adenosine 5′ monophosphate
3′,5′ cNMP	3′,5′ cyclic nucleotide
nt	nucleotide
pG	5′ guanylic acid
pGpG	5′ diguanylic acid
pGp	5′ guanylic acid 3′ phosphate
pC	5′ cytidylic acid
pCpC	5′ dicytidylic acid
pCp	5′ cytidylic acid 3′ phosphate
DBU	1,8-diazabicyclo(5.4.0)undec-7-ene
MALDI	matrix-assisted laser desorption/ionization
ToF	time of flight
ESI	electrospray ionization
MS	mass spectrometry
HPLC	high-performance liquid chromatography
